# A Multilevel Analysis of Regressors of Access to Improved Drinking Water and Sanitation Facilities in Ghana

**DOI:** 10.1155/2019/3983869

**Published:** 2019-06-04

**Authors:** Pascal Agbadi, Ernest Darkwah, Paul L. Kenney

**Affiliations:** ^1^Department of Nursing, Faculty of Allied Health Sciences, College of Health Sciences, Kwame Nkrumah University of Science and Technology, Kumasi, Ghana; ^2^Department of Psychology, University of Ghana, P.O.Box LG 84, Legon, Ghana; ^3^Institute of Statistical, Social and Economic Research, University of Ghana, P.O.Box LG74, Legon-Accra, Ghana

## Abstract

People's access to quality water and sanitation resources significantly improves their health. Using the 2014 Ghana DHS dataset, multilevel robust Poisson regression modelling was performed to investigate the factors that enhance Ghanaian households' access to improved sources of drinking water and toilet facilities. The results indicated that household head and household socioeconomic factors have significant effects on access to improved sources of drinking water and toilet facilities, and this varies from one community of residence to another. The following households had a higher probability of having access to improved sources of drinking water: female-headed households, households with heads who had at least attained middle-school-level education, urban households, and nonpoorest households. Correspondingly, the following households were more likely to have access to improved toilet facilities: female-headed households had a higher chance of access, as well as those whose heads had at least middle-school-level education, were at least 35 years old, or were currently married, rural households, households with a minimum of seven members, and households who attained at least middle wealth status. In their efforts to increase citizens' access to improved water and sanitation facilities, the government and other development organizations should develop citizens' wealth-creation capacities and enable their attainment of formal education.

## 1. Introduction

Safe water and sanitation are prerequisites of health, human growth, and development. Thus, promoting and ensuring people's access to improved sources of water and sanitation facilities are both public health promotion and human right agenda [1, 2]. Unhygienic sources of drinking water and unhealthy sanitation facilities result in skin diseases, acute respiratory infections (ARIs), diarrheal diseases, Guinea worm disease, typhoid, cholera, schistosomiasis, trachoma, dysentery, death, etc. [3–6].

In 2015, unsafe water, sanitation, and hygiene (unWASH) practices resulted in about 1,766,000 global deaths and about 95,305,000 global disability-adjusted life years (DALYs) [7]. The same conditions resulted in about 7,300 deaths and 435,500 DALYs in Ghana in 2015 [7]. In Ghana, mortality and morbidity attributable to unWASH in children and adults are stabilizing, but unWASH practices remain common causes of death and morbidity in the country. In Ghana, about 87% of the population has access to safe drinking water, about 15% has access to improved toilet facilities, and about 20% practice open defecation [8].

Existing literature indicates that a plethora of social, economic, political, and environmental factors explain the differences in access to improved sources of drinking water and toilet facilities around the world. Studies have suggested that the region of residence, locality (urban/rural) of residence, household size, household wealth status, and the household head's characteristics (such as gender, age, level of education, marital status, and employment status) are significantly related to the household's access to improved water and sanitation facilities in places such as Zambia [9], Nigeria [10], Bhutan [11], Indonesia [6, 12, 13], Timor-Leste [14], Kenya [15], and Ethiopia [16].

Data on the prevalence of the debilitating effects of unWASH in Ghana are within reach, but only a few publications have investigated the socioeconomic structures that increase Ghanaian residents' access to quality water sources and sanitation infrastructures [17–19]. The only study that used nationally representative data (2008 DHS data) did not adopt a statistical approach that accounts for the hierarchical nature of the data. Ignoring clustering in analyzing data could result in underestimation of parameters [20]. Using the 2014 Ghana Demographic and Health Survey dataset, we performed a multilevel robust Poisson regression modelling in order to illuminate prevalence ratio which is preferred over the odds ratio for most cross-sectional studies [21–24]. Taking the community of residence as a level 2 unit, we sought to answer the following questions: (i) What factors predict access to improved sources of drinking water for Ghanaian households? (ii) What factors predict access to improved toilet facilities for Ghanaian households?

## 2. Materials and Methods

### 2.1. Design

The 2014 Ghana Demographic and Health Survey (GDHS) dataset used in this paper was collected in accordance with cross-sectional design protocols [8]. The 2014 GDHS was implemented by the Ghana Statistical Service (GSS), the Ghana Health Service (GHS), and the National Public Health Reference Laboratory (NPHRL) of the GHS [8]. The survey employed two-staged probability sampling in the ten administrative regions of Ghana [8].

The 2014 GDHS used an updated sampling frame from the 2010 Ghana Population and Housing Census acquired from the Ghana Statistical Service [8]. The first stage of the probability sampling involved the selection of enumeration areas (EAs) [8]. A total of 427 EAs were selected, 216 in urban areas and 211 in rural areas [8]. The second stage of the probability sampling involved the systematic sampling of households [8]. The implementers of the survey undertook a household-listing operation in all the selected EAs from January through March 2014, and households to be included in the survey were randomly selected from the list [8]. Approximately 30 households were selected from each EA to constitute a total sample size of 12,831 households [8].

### 2.2. Data Collection

Data collection was done by trained enumerators from early September to mid-December 2014 using paper-based questionnaires [8]. The selected sample size for the 2014 GDHS was 12,831 households, of which 12,010 were occupied [8]. Out of the occupied households, 11,835 were successfully interviewed, resulting in a response rate of 99 percent [8].

### 2.3. Study Area

The study area is Ghana. Ghana returned to multiparty democracy in 1992 and has since enjoyed relative political stability and peace. Ghana's population is about 29 million. Accra, in the Greater Accra Region, is the administrative and political capital city of Ghana. Ghana has embarked on many programs of industrialization, economic growth and development, education, health, and poverty alleviation that have attracted funding from international governments and multinational agencies. In the past three decades, many of these economic development programs have reached all the regions of Ghana through decentralization and local governance reforms. The country has made some significant economic gains by coming out of a highly indebted poor country (HIPC) status to its current lower-middle income. Ghana's national estimates of the adult literacy rate, poverty level, doctor-to-patient ratio are 56%, 24%, and one doctor to 800 patients, respectively [25]. The relatively good national estimates, however, mask the prevailing socioeconomic inequalities between urban and rural areas and among the ten administrative regions in Ghana [25]. Regions in the north of Ghana are economically marginalized compared to the regions in the south. Also, economic disparities exist between rural and urban areas of the country. About 38% of Ghanaians in rural areas are poor compared to about 11% urban poor [25]. The three regions—Upper West, Upper East, and Northern—in the north of Ghana are much poorer compared to the regions in the south. Out of 6,384,058 poor people in Ghana, about 2,337,591 (representing 37%) are found in the three northern regions of Ghana [25]. The Greater Accra Region, with a poverty rate of 3.8, has the least number of poor people in Ghana [25].

Rural-urban disparities in access to improved sources of drinking water have been reported. About 93% of the urban population have access to improved sources of drinking water compared to 84% rural dwellers [26]. In a report by Nana Yaw and Sarah [27], it is noted that about 20% of the entire country's population practice open defecation. The practice is much more prevalent in the three regions of Northern Ghana—Northern, Upper East, and Upper West—where more than 70% of the population practice open defecation [27]. The report further noted that almost 51% of Ghanaians use communal latrines which, according to the Joint Monitoring Programme of WHO/UNICEF, are classified as unimproved [27].

### 2.4. Study Sample

The unit of analysis is households, and the number of cases in the dataset was 11,835 households. Six cases had missing information in some of the variables and were excluded from the analysis; thus, there were 11,829 analytic samples. In the GDHS, household heads provided information on their demographic characteristics and household characteristics such as household population and composition, housing structure, household assets, access to basic utilities, sources of drinking water, water treatment practices, access to sanitation facilities, and type of fuel used for cooking [8].

### 2.5. Variables

The variables used in the study have been listed and described in Table 1.

### 2.6. Data Analysis

Statistical analyses were performed in R freeware version 3.5.3 [28] and SAS software version 9.4 [29]. Statistical significance was pegged at thresholds of 1% and 5% (*p* ≤ 0.01 and *p* ≤ 0.05). The pastecs and gmodels packages in R were used to perform summary statistics and bivariate analyses, respectively. The lme4 package was used to model the multilevel binary models, and the sjstats packages were used to determine their intercluster correlation coefficients. The SAS PROC GENMOD with the specification of SUBJECT = cluster identifier in the REPEATED statement was used to perform the two-level multilevel Poisson regression modelling by the use of the sandwich variance estimator [24]. Models that fit with the REPEATED statement use the generalized estimating equation (GEE) method to estimate the model. Thus, the quasi-likelihood information criterion (QIC), instead of the popular Akaike's information criterion (AIC), is used to evaluate the goodness of fit of the models because the GEE method is not a likelihood-based method [30]. SAS version 9.4 provides the QIC value along with results in the output. Basically, the model with the smallest QIC is the best fitting model.

### 2.7. Binary Outcome Multilevel Modelling

Due to the stratified nature of data in the 2014 GDHS, the households are nested into communities of residence. To account for the hierarchically clustered nature of the survey dataset, multilevel robust Poisson models were generated to avoid possible underestimation of parameters from a single-level model and the potential overestimation of effects from reporting odds ratios when the prevalence rate is above 10% [20, 21, 24, 31]. The community of residence was chosen as the level 2 variable under which the households (level 1 variable) are nested. Two multilevel robust Poisson regression modellings were performed to assess the impact of household head factors and household socioeconomic factors on a household's access to an improved source of drinking water and improved toilet facilities.

Four models were tested in each of the cases (access to an improved source of drinking water and access to improved toilet facilities). Model 0 is the null model, which assessed the percentage variation in access to improved sources of drinking water and improved toilet facilities explained by the level 2 units (community of residence). Model 1 investigated the impact of demographic characteristics of household heads on the likelihood of having access to improved sources of drinking water and improved toilet facilities. Model 2 assessed the impact of household socioeconomic factors on the likelihood of having access to improved sources of drinking water and improved toilet facilities. Model 3 assessed the impact of household head factors and household socioeconomic factors on access to improved sources of drinking water and improved toilet facilities. The association between the criterion variables and the explanatory variables was measured in adjusted prevalence ratio (aPR) and its 95% confidence interval (CI).

Assumptions of collinearity were checked, and no violations were observed; all explanatory variables in the models have the values of generalized variance inflation factors (VIFs) less than 4 which is far less than the cutoff value of 10 in [32] (see Table 2).

### 2.8. How the Criterion Variables Were Handled in the Analyses?

For the first research question, the criterion variable denotes *access to improved sources of drinking water*, which takes the value 0 or 1 for each household (0 = unimproved sources of drinking water; 1 = improved sources of drinking water).

For the second research question, the criterion variable denotes *access to improved toilet facilities*, which takes the value 0 or 1 for each household (0 = unimproved toilet facilities; 1 = improved toilet facilities).

### 2.9. Ethical Considerations

The 2014 GDHS protocol was reviewed and approved by the Ghana Health Service Ethical Review Committee and the Institutional Review Board of ICF International. Informed consent was also obtained from participants before they were interviewed [8]. The GDHS is publicly available upon a simple, registration-access request, so no further ethical clearance was sought.

## 3. Results

### 3.1. Sample Characteristics

A total of 1,415 households and 10,406 households have access to improved toilet facilities (12%) and improved sources of drinking water (88%), respectively. Most households are headed by males (68%). Most respondents have attained at least the basic school level of education—middle/JSS/JHS (34%). Most household heads were currently married (63%). The remaining sample characteristics are reported in Table 3.

### 3.2. Test of Association between the Criterion Variables and the Explanatory Variables

The chi-square test of independence was performed to explore (i) the relationship between households' sources of drinking water (SDW) and categorical explanatory variables (Table 4) and (ii) the relationship between types of toilet facilities used by households and categorical explanatory variables (Table 5).

### 3.3. Correlates of Improved Sources of Drinking Water

The analyses revealed that there was a statistically significant relationship between a household source of drinking water (SDW) and all the categorical explanatory variables (Table 4). Only about 1,293 households (12.4%) had access to both improved sources of drinking water and improved toilet facilities.

### 3.4. Correlates of Improved Toilet Facilities

Table 5 reports the results of the chi-square test of independence between the types of toilet facilities (TTF) used by households and categorical explanatory variables. The analyses revealed that there was a statistically significant relationship between TTF used by households and all the categorical explanatory variables (Table 5).

### 3.5. Regressors of Access to Improved Sources of Drinking Water

Table 6 presents the adjusted prevalence ratios (aPRs) and confidence intervals (CIs) of a multilevel robust Poisson regression model delineating the predictors of households' access to improved sources of drinking water (ISDW) in Ghana. The following households were more likely to have access to improved sources of drinking water in Ghana: households with female heads, households with heads who at least have a middle-school-level education, urban residence households, and households that were nonpoorest (Table 6, Model 3). The ICC value of 0.76 indicates that 76% of the total variation in the criterion variable is accounted for by the community the households are located. Thus, the remaining 24% variability is due to the variation within the households and other unknown factors.

Compared to male-headed households, female-headed households had a higher probability of access to improved sources of drinking water (aPR: 1.01; 95% CI: 1.00–1.03). Compared to households with heads without formal education, households with heads who attained middle/JSS/JHS-level education (aPR: 1.02; 95% CI: 1.00–1.04) or secondary/SSS/SHS-level education (aPR: 1.02; 95% CI: 1.00–1.04) or postsecondary-school-level education (aPR: 1.03; 95% CI: 1.00–1.05) had a higher chance of having access to improved sources of drinking water. Compared to rural households, urban households had a higher probability of having access to the improved drinking water source by 10% (aPR: 1.10; 95% CI: 1.04–1.15). Compared to poorest households' access to improved sources of drinking water, households with the following wealth indices have the following positive implications: poor households had a 6% higher probability (aPR: 1.06; 95% CI: 1.02–1.10), middle households had a 16% higher probability (aPR: 1.16; 95% CI: 1.10–1.21), richer households had an 18% higher probability (aPR: 1.18; 95% CI: 1.12–1.23), and richest households had an 18% higher probability (aPR: 1.18; 95% CI: 1.13–1.24).

### 3.6. Regressors of Access to Improved Toilet Facilities

Table 7 presents the adjusted prevalence ratios (aPRs) and confidence intervals (CIs) of a multilevel robust Poisson regression model outlining the predictors of households' access to improved toilet facilities (ITF) in Ghana. The following households were more likely to have access to improved toilet facilities: rural households, households with a minimum of seven members, and households who have at least middle wealth status (Table 7, Model 2). When household head factors were accounted for in Models 2 and 3, it is clear that female-headed households, households with heads of at least 35 years old, and households with heads who attained at least a middle-school-level education increased the households' probability of having access to an improved type of toilet facility. The ICC value of 0.26 indicates that 26% of the total variation in the criterion variable is accounted for by the community the households are located. Thus, the remaining 74% variability is due to the variation within the households and other unknown factors.

In Model 2, compared to rural households, urban households had a lower probability of having access to improved toilet facilities by 38% (aPR: 0.62; 95% CI: 0.50–0.77). Compared to households with at least 7 members, households with 4–6 members (aPR: 0.77; 95% CI: 0.65–0.85) and households with 1–3 members (aPR: 0.48; 95% CI: 0.42–0.55) had a lower likelihood of having access to improved toilet facilities. Compared to poorest households' access to improved toilet facilities, households with the following wealth indices have the following positive implications: middle households had an 81% higher likelihood (aPR: 1.81; 95% CI: 1.37–2.34), richer households had a 219% higher probability (aPR: 3.19; 95% CI: 2.40–4.25), and richest households had an 844% higher probability (aPR: 9.44; 95% CI: 7.08–12.61).

## 4. Discussion

This section discusses the results of the study presented above. The aim of the study was to investigate factors associated with access to improved sources of drinking water and toilet facilities in Ghana. We found that certain household head and household socioeconomic factors have significant effects on access to improved sources of drinking water and toilet facilities. However, the effects of such factors vary at the community level. This implies that the type of community a household is located in has an influence on its access to sanitation resources. We also observed a high prevalence in access to improved sources of drinking water. In all Ghanaian communities, public tap/standpipe, tube well/borehole, and purified sachet water, classified as improved sources of drinking water by the Joint Monitoring Programme of WHO/UNICEF, are within reach and are affordable. This suggests that general availability and provision of both quality water sources and toilet facilities will influence households' access.

The study again observed that the gender of the household head was a statistically significant predictor of the household's access to improved sources of drinking water and improved toilet facilities. More specifically, compared to male-headed households, female-headed households had a higher probability of having access to improved sources of drinking water and improved toilet facilities. One possible explanation for this result could be that relative to men, most sub-Saharan African women have a greater household responsibility (such as cooking, cleaning, and laundry) which requires heavy use of water. Thus, it is plausible that female heads, to reduce the burden that comes with fetching water from far distanced places, will ensure that the households they are heading have good access to water and sanitation facilities. The results that we obtained here are consistent with those of previous studies, which found that female-headed households were more likely to have access to improved sources of drinking water and sanitation facilities [9–12, 14, 17].

The household head's education level was statistically significantly associated with the likelihood of having access to an improved source of drinking water and toilet facilities. Households with heads with no formal education had a lower probability of having access to improved sources of drinking water and toilet facilities. Generally, education is a resource factor of quality health outcomes in communities in sub-Saharan Africa. This is because educated people are usually more aware of conditions that guarantee their well-being, and they could have easier access to resources that can create healthy conditions around them. This implies that educated household heads in this study may have utilized their resources to provide their households with improved water and toilet facilities. These results are in consonance with the results of studies with a similar scope [10–13, 17, 19].

In the model for toilet facility access, the age of the household head predicted the likelihood of having access to improved toilet facilities but not access to improved sources of drinking water. Households with heads who were at least 35 years old had a higher chance of having access to improved toilet facilities. This suggests that as people age, they become more conscious of their health and may desire to utilize facilities that improve their quality of life. Although undocumented in the literature, it is suggestive to argue that old age in most African communities is associated with respect and civility. Therefore, individuals of at least 35 years may be morally compelled to provide improved sanitation facilities in order to avoid the public ridicule that is often associated with open defecation. Also, anecdotal evidence suggests that citizens within this age group are more likely to have some form of income source or employment which likely makes them able to afford utilities. While this finding from our study confirms earlier findings that the age of the household head is a predictor of the household's access to an improved source of drinking water [11], it contradicts findings from other investigations that found no associations between these variables [10].

In Model 3 of access to improved toilet facilities, households with heads who were currently married at the time of the survey had a higher probability of having access to improved toilet facilities, but this had no effect on their access to improved sources of drinking water. The protective effect of the household head being married against the lack of access to improved toilet facilities is consistent with the findings of other studies [15]. However, the study's results are in contrast with those of other studies with regard to household heads who were not currently married. One study, using the 2008 Ghana Demographic and Health Survey, found that households with heads who had never married were more likely to have had access to improved toilet facilities [17]. Also, while the study found no predictive effect of the marital status of household heads on the likelihood of having access to improved sources of drinking water, it was observed in other studies [12, 17]. The implication is that contextual differences exist regarding access to sanitation resources and the factors influencing such access.

Urban households were more likely to have access to improved sources of drinking water, but rural households were more likely to have access to improved toilet facilities. These results are in consonance with the results of other researchers who found that urban households were more likely to have access to improved sources of drinking water [9, 11–14, 17, 19]. In this study and those of others [9, 14], an increase in the probability of access to improved toilet facilities was observed for rural households contrary to the expectation. This brings an interesting twist to popular discourses about the nature of unequal access to utilities between urban and rural settings as the view has often been that urban dwellers have better access [33]. The protective effect of rural household's access to an improved type of toilet facility could be attributed to the positive contributions of both local and international NGO activities in rural communities on sanitation [34]. However, with regard to access to toilet facilities, some studies found urban households to have had a higher chance of access [13, 17].

Households with more members were more likely to have access to improved toilet facilities but not access to improved drinking water. Other studies also found that larger households were more likely to have access to improved toilet facilities [17]. This finding could mean that the larger the households, the more the resources they may have for building improved toilet facilities. Unlike this study's results, other studies indicated that household size predicted the likelihood of having access to improved sources of drinking water [12, 17].

Nonpoorest households had a greater probability of having access to both improved sources of drinking water and improved toilet facilities. Similar to the findings of this study, other studies found that household wealth is a major factor in explaining the differences in the probability of having access to improved sources of drinking water and toilet facilities [9–14, 16, 17, 19]. The convergence of findings from different contexts on this subject strongly suggests that poverty is a major risk factor that prevents many households from accessing improved water and sanitation facilities.

## 5. Limitations and Strengths

This study used data from a cross-sectional survey; therefore, the results from analyzing the data cannot establish causal relationships. The conclusions in this study are restricted to associations between the explanatory and the criterion variables. Besides the limitation, there is one strength of the study. The study used the GDHS dataset that is nationally representative and large. Therefore, findings from the analyses can be generalized for the entire Ghanaian population. The GDHS programs have consistently been undertaken about 7 times already since the 1980s; thus, their sampling methodologies, data collection procedures, and questionnaires have been improved.

## 6. Conclusions

This study sought to investigate the predictive factors of the household's access to improved sources of drinking water and improved toilet facilities in Ghana. About 88% of households had access to improved sources of drinking water and 12% to improved toilet facilities. The effects of household head and household socioeconomic factors on access to improved sources of drinking water and toilet facilities vary from one community of residence to another. The following households had a higher probability of access to improved sources of drinking water: households with female heads, households with heads who have at least middle-school-level education, urban residence households, and households that were nonpoorest. With regard to improved toilet facilities, female-headed households had a higher chance of access, as well as those whose heads had at least middle-school-level education, were at least 35 years old, or were currently married. Also, rural households, those with a minimum of seven members, and those with at least middle wealth status had a higher chance of access to improved toilet facilities.

Household wealth was an extremely significant factor in determining access to improved sources of drinking water and toilet facilities. Thus, the authors recommend that the government of Ghana and her development partners should promote policies and programs that enhance citizens' wealth creation capacities. The authors recommend that investments in improving access to water and sanitation facilities in Ghana should be complemented with programs that encourage citizens to obtain a formal education. It is common knowledge that increasing the actual quantity of functional facilities available in a community could greatly influence the households' access. The authors, therefore, recommend that state authorities and nongovernmental organizations pursue policies and intervention that aim at increasing the units of improved toilet facilities and drinking water sources in all communities, taking into account the variations in access attributed to the context of the community where the households were located.

## Figures and Tables

**Table 1:** Criterion and explanatory variables.

Variables	Descriptions
*Criterion variables*	
Sources of drinking water	(i) Improved: a household is said to have access to an improved drinking water source if it has water piped into its dwelling, water piped to a yard/plot, a public tap/standpipe, a tube well/borehole, a protected dug well, a protected spring, rainwater, bottled water, or sachet water
	(ii) Unimproved: a household is said to have access to an unimproved drinking water source if it has an unprotected dug well, an unprotected spring, a tanker truck/cart with a small tank, or surface water
Types of toilet facilities	(i) Improved: a household is said to have access to improved toilet facilities if it has unshared flush/pour flush to piped sewer systems, septic tanks or pit latrines, ventilated improved pit latrines, composting toilets, or pit latrines with slabs
	(ii) Unimproved: a household is said to have access to unimproved toilet facilities if it has improved facilities that are shared, flush/pour flush not to sewer/septic tank/pit latrine, pit latrine without slab/open pit, bucket, hanging toilet/hanging latrine, or no facility/bush/field

*Explanatory variables*	
Sex of the household head	(i) Male (ii) Female
Age of the household head	(i) 15–34 years (ii) 35–54 years (iii) 55 years and above
Education level of the household head	(i) No education/preschool (ii) Primary (iii) Middle/JSS/JHS (iv) Secondary/SSS/SHS (v) Postsecondary
Marital status of the household head	(i) Never married/never lived together (ii) Currently married (iii) Formerly/ever married
Household wealth index	(i) Poorest (ii) Poor (iii) Middle (iv) Richer (v) Richest
Household size	(i) 1–3 (ii) 4–6 (iii) 7+
Place of residence of household	(i) Rural (ii) Urban
Region of residence of household	(i) Greater Accra (ii) Eastern (iii) Volta (iv) Central (v) Western (vi) Ashanti (vii) Brong Ahafo (viii) Northern (ix) Upper East (x) Upper West
Primary sampling unit	Handled as a community in the study

**Table 2:** Collinearity statistics of explanatory variables.

Explanatory variables	Df	Improved water model		Improved toilet model	
		GVIF	GVIF^(1/(2^*∗*^Df))^	GVIF	GVIF^(1/(2^*∗*^Df))^
Sex of the HH	1	1.56	1.25	1.49	1.22
Age of the HH	2	1.49	1.10	1.61	1.13
Education level of the HH	4	1.42	1.04	1.53	1.05
Marital status of the HH	2	2.21	1.22	2.29	1.23
Place of residence	1	1.06	1.03	1.49	1.22
Household size	2	1.34	1.08	1.32	1.07
Household wealth	2	1.30	1.03	1.98	1.09
Improved source of water	1			1.09	1.04
An improved type of toilet	1	1.03	1.02		

GVIF: generalized variance inflation factor; Df: degree of freedom.

**Table 3:** Descriptive analysis of variables (*N* = 11,829).

Variables	*N*	%
*Sources of drinking water*		
Unimproved	1429	12
Improved	10,400	88

*Toilet facilities*		
Unimproved	10,414	88
Improved	1415	12

*Sex of the household head*		
Female	3825	32
Male	8004	68

*Age of the household head*		
15–34 years	3691	31
35–54 years	4876	41
55 years and above	3262	28

*Education level of the household head*		
No education/preschool	3346	28
Primary	1662	14
Middle/JSS/JHS	4082	35
Secondary/SSS/SHS	1511	13
Postsecondary	1228	10

*Marital status of the household head*		
Never married/never lived together	1756	15
Currently married	7454	63
Formerly/ever married	2619	22

*Household size*		
1–3 members	6267	53
4–6 members	4079	35
7 members and above	1483	13

*Household wealth index*		
Poorest	2508	21
Poor	2418	20
Middle	2577	22
Richer	2285	19
Richest	2041	17

*Place of residence*		
Rural	5894	50
Urban	5935	50

*Region of residence*		
Northern	1014	9
Central	1228	10
Greater Accra	1337	11
Volta	1115	9
Eastern	1307	11
Ashanti	1310	11
Brong Ahafo	1325	11
Western	1302	11
Upper East	998	8
Upper West	893	8

*Note*. Due to rounding errors, percentages may not equal 100%.

**Table 4:** Bivariate analyses of SDW and explanatory variables.

	Source of drinking water (SDW)	
	Unimproved SDW	Improved SDW
*Types of toilet facilities*		
Unimproved toilet facilities	1307 (91.5%)	9107 (87.6%)
Improved toilet facilities	122 (8.5%)	1293 (12.4%)
*p* ≤ 0.001; *χ* ^2^ = 17.73^*∗*^; df = 1		

*Sex of the household head*		
Female	327 (22.9%)	3498 (33.6%)
Male	1102 (77.1%)	6902 (66.4%)
*p* ≤ 0.001; *χ* ^2^ = 66.38^*∗*^; df = 1		

*Age of the household head*		
15–34 years	379 (26.5%)	3312 (31.8%)
35–54 years	594 (41.6%)	4282 (41.2%)
55 years and above	456 (31.9%)	2806 (27.0%)
*p* ≤ 0.001; *χ* ^2^ = 22.53; df = 2		

*Education level of the household head*		
No education/preschool	618 (43.2%)	2728 (26.2%)
Primary	280 (19.6%)	1382 (13.3%)
Middle/JSS/JHS	415 (29.0%)	3667 (35.3%)
Secondary/SSS/SHS	85 (5.9%)	1426 (13.7%)
Postsecondary	31 (2.2%)	1197 (11.5%)
*p* ≤ 0.001; *χ* ^2^ = 343.10; df = 4		

*Marital status of the household head*		
Never married/never lived together	123 (8.6%)	1633 (15.7%)
Currently married	1017 (71.2%)	6437 (61.9%)
Formerly/ever married	289 (20.2%)	2330 (22.4%)
*p* ≤ 0.001; *χ* ^2^ = 62.44; df = 2		

*Household size*		
1–3 members	629 (44.0%)	5638 (54.2%)
4–6 members	513 (35.9%)	3566 (34.3%)
7 members and above	287 (20.1%)	1196 (11.5%)
*p* ≤ 0.001; *χ* ^2^ = 99.43; df = 2		

*Household wealth index*		
Poorest	655 (45.8%)	1853 (17.8%)
Poor	551 (38.6%)	1867 (18.0%)
Middle	175 (12.2%)	2402 (23.1%)
Richer	42 (2.9%)	2243 (21.6%)
Richest	6 (0.4%)	2035 (19.6%)
*p* ≤ 0.001; *χ* ^2^ = 1286.00; df = 4		

*Place of residence*		
Rural	1192 (83.4%)	4702 (45.2%)
Urban	237 (16.6%)	5698 (54.8%)
*p* ≤ 0.001; *χ* ^2^ = 731.95^*∗*^; df = 1		

*Region of residence*		
Northern	251 (17.6%)	763 (7.3%)
Central	118 (8.3%)	1110 (10.7%)
Greater Accra	32 (2.2%)	1305 (12.6%)
Volta	195 (13.6%)	920 (8.8%)
Eastern	231 (16.2%)	1076 (10.3%)
Ashanti	97 (6.8%)	1213 (11.7%)
Brong Ahafo	169 (11.8%)	1156 (11.1%)
Western	195 (13.6%)	1107 (10.6%)
Upper East	97 (6.8%)	901 (8.7%)
Upper West	44 (3.1%)	849 (8.2%)
*p* ≤ 0.001; *χ* ^2^ = 433.80; df = 9		

*Note.* ^*∗*^Yates' continuity correction. Column percentages are reported.

**Table 5:** Bivariate analyses of types of toilet facilities and explanatory variables.

	Types of toilet facilities (TTF)	
	Unimproved TTF	Improved TTF
*Sex of the household head*		
Female	3408 (32.7%)	417 (29.5%)
Male	7006 (67.3%)	998 (70.5%)
*p* ≤ 0.05; *χ* ^2^ = 6.03^*∗*^; df = 1		

*Age of the household head*		
15–34 years	3422 (32.9%)	269 (19.0%)
35–54 years	4216 (40.5%)	660 (46.6%)
55 years and above	2776 (26.7%)	486 (34.3%)
*p* ≤ 0.001; *χ* ^2^ = 114.7; df = 1		

*Education level of the household head*		
No education/preschool	3133 (30.1%)	213 (15.1%)
Primary	1548 (14.9%)	114 (8.1%)
Middle/JSS/JHS	3618 (34.7%)	464 (32.8%)
Secondary/SSS/SHS	1283 (12.3%)	228 (16.1%)
Postsecondary	832 (8.0%)	396 (28.0%)
*p* ≤ 0.001; *χ* ^2^ = 635.84; df = 4		

*Marital status of the household head*		
Never married/never lived together	1612 (15.5%)	144 (10.2%)
Currently married	6441 (61.8%)	1013 (71.6%)
Formerly/ever married	2361 (22.7%)	258 (18.2%)
*p* ≤ 0.001; *χ* ^2^ = 53.43; df = 2		

*Household size*		
1–3 members	5627 (54.0%)	640 (45.2%)
4–6 members	3512 (33.7%)	567 (40.1%)
7 members and above	1275 (12.2%)	208 (14.7%)
*p* ≤ 0.001; *χ* ^2^ = 38.77; df = 2		

*Household wealth index*		
Poorest	2370 (22.8%)	138 (9.8%)
Poor	2249 (21.6%)	169 (11.9%)
Middle	2385 (22.9%)	192 (13.6%)
Richer	2052 (19.7%)	233 (16.5%)
Richest	1358 (13.0%)	683 (48.3%)
*p* ≤ 0.001; *χ* ^2^ = 1108.7; df = 4		

*Place of residence*		
Rural	5398 (51.8%)	496 (35.1%)
Urban	5016 (48.2%)	919 (64.9%)
*p* ≤ 0.001; *χ* ^2^ = 139.65^*∗*^; df = 1		

*Region of residence*		
Northern	968 (9.3%)	46 (3.3%)
Central	1090 (10.5%)	138 (9.8%)
Greater Accra	1035 (9.9%)	302 (21.3%)
Volta	954 (9.2%)	161 (11.4%)
Eastern	1121 (10.8%)	186 (13.1%)
Ashanti	1124 (10.8%)	186 (13.1%)
Brong Ahafo	1209 (11.6%)	116 (8.2%)
Western	1123 (10.8%)	179 (12.7%)
Upper East	953 (9.2%)	45 (3.2%)
Upper West	837 (8.0%)	56 (4.0%)
*p* ≤ 0.001; *χ* ^2^ = 313.15; df = 9		

*Note.* ^*∗*^Yates' continuity correction. Column percentages are reported.

**Table 6:** Predictors of access to improved sources of drinking water.

Explanatory variables	Model 0 aPR (95% CI)	Model 1 aPR (95% CI)	Model 2 aPR (95% CI)	Model 3 aPR (95% CI)
*Sex of the household head*				
Male		1.00 (reference)		1.00 (reference)
Female		1.02^*∗∗*^ (1.01, 1.03)		1.01^*∗*^ (1.00, 1.03)

*Age of the household head*				
15–34 years		1.00 (reference)		1.00 (reference)
35–54 years		1.00 (0.99, 1.02)		1.01 (0.99, 1.02)
55 years and above		1.00 (0.99, 1.02)		1.01 (1.00, 1.03)

*Education level of the household head*				
No education/preschool		1.00 (reference)		1.00 (reference)
Primary		1.03^*∗∗*^ (1.01, 1.05)		1.02 (1.00, 1.04)
Middle/JSS/JHS		1.04^*∗∗*^ (1.02, 1.06)		1.02^*∗*^ (1.00, 1.04)
Secondary/SSS/SHS		1.05^*∗∗*^ (1.03, 1.07)		1.02^*∗*^ (1.00, 1.04)
Postsecondary		1.07^*∗∗*^ (1.05, 1.10)		1.03^*∗*^ (1.01, 1.05)

*Marital status of the household head*				
Never married nor lived together		1.00 (reference)		1.00 (reference)
Currently married		0.99 (0.97, 1.00)		1.00 (0.98, 1.01)
Formerly/ever married		0.99 (0.98, 1.01)		0.99 (0.98, 1.01)

*Toilet facilities*				
Unimproved			1.00 (reference)	1.00 (reference)
Improved			0.99 (0.97, 1.01)	0.99 (0.97, 1.01)

*Place of residence*				
Rural			1.00 (reference)	1.00 (reference)
Urban			1.10^*∗∗*^ (1.05, 1.16)	1.10^*∗∗*^ (1.04, 1.15)

*Household size*				
7 members and above			1.00 (reference)	1.00 (reference)
4–6 members			1.00 (0.98, 1.02)	1.00 (0.98, 1.02)
1–3 members			1.01 (0.99, 1.03)	1.01 (0.99, 1.03)

*Household wealth index*				
Poorest			1.00 (reference)	1.00 (reference)
Poor			1.06^*∗∗*^ (1.02, 1.11)	1.06^*∗∗*^ (1.02, 1.10)
Middle			1.16^*∗∗*^ (1.11, 1.22)	1.16^*∗∗*^ (1.10, 1.21)
Richer			1.19^*∗∗*^ (1.13, 1.24)	1.18^*∗∗*^ (1.12, 1.23)
Richest			1.19^*∗∗*^ (1.14, 1.25)	1.18^*∗∗*^ (1.13, 1.24)
Intercept (community)	0.88^*∗∗*^ (0.86, 0.90)	0.85^*∗∗*^ (0.82, 0.88)	0.77^*∗∗*^ (0.70, 0.79)	0.73^*∗∗*^ (0.68, 0.78)

*Random effects*				
Observations	11829	11829	11829	11829
Second-level units (community)	427	427	427	427
ICC	0.88	0.81	0.76	0.76
QIC	194101.25	195298.46	195294.11	195111.03
QICu	194072.76	195277.41	195240.20	195065.67

^*∗∗*^ *p* ≤ 0.01; ^*∗*^ *p* ≤ 0.05; ICC: intracommunity correlation coefficient; QIC: quasi-likelihood information criterion; QICu: the simplified version of the QIC.

**Table 7:** Predictors of access to improved toilet facilities.

Explanatory variables	Model 0 aPR (95% CI)	Model 1 aPR (95% CI)	Model 2 aPR (95% CI)	Model 3 aPR (95% CI)
*Sex of the household head*				
Male		1.00 (reference)		1.00 (reference)
Female		1.20^*∗∗*^ (1.06, 1.25)		1.18^*∗∗*^ (1.05, 1.33)

*Age of the household head*				
15–34 years		1.00 (reference)		1.00 (reference)
35–54 years		1.88^*∗∗*^ (1.60, 2.21)		1.70^*∗∗*^ (1.45, 2.00)
55 years and above		2.62^*∗∗*^ (2.21, 3.11)		2.60^*∗∗*^ (2.18, 3.10)

*Education level of the household head*				
No education/preschool		1.00 (reference)		1.00 (reference)
Primary		1.14 (0.96, 1.35)		1.12 (0.93, 1.36)
Middle/JSS/JHS		1.47^*∗∗*^ (1.26, 1.70)		1.19^*∗*^ (1.01, 1.41)
Secondary/SSS/SHS		2.16^*∗∗*^ (1.79, 2.59)		1.45^*∗∗*^ (1.20, 1.75)
Postsecondary		3.77^*∗∗*^ (3.18, 4.48)		2.12^*∗∗*^ (1.74, 2.58)

*Marital status of the household head*				
Never married nor lived together		1.00 (reference)		1.00 (reference)
Currently married		1.61^*∗∗*^ (1.32, 1.96)		1.31^*∗∗*^ (1.09, 1.56)
Formerly/ever married		1.12 (0.89, 1.41)		1.07 (0.87, 1.33)

*Sources of drinking water*				
Unimproved			1.00 (reference)	1.00 (reference)
Improved			0.82 (0.62, 1.09)	0.82 (0.62, 1.07)

*Place of residence*				
Rural			1.00 (reference)	1.00 (reference)
Urban			0.62^*∗∗*^ (0.50, 0.77)	0.63^*∗∗*^ (0.52, 0.77)

*Household size*				
7 members and above			1.00 (reference)	1.00 (reference)
4–6 members			0.77^*∗∗*^ (0.65, 0.85)	0.80^*∗∗*^ (0.70, 0.92)
1–3 members			0.48^*∗∗*^ (0.42, 0.55)	0.59^*∗∗*^ (0.51, 0.69)

*Household wealth index*				
Poorest			1.00 (reference)	1.00 (reference)
Poor			1.21 (0.92, 1.59)	1.19 (0.90, 1.54)
Middle			1.81^*∗∗*^ (1.37, 2.34)	1.77^*∗∗*^ (1.33, 2.34)
Richer			3.19^*∗∗*^ (2.40, 4.25)	2.90^*∗∗*^ (2.14, 3.93)
Richest			9.44^*∗∗*^ (7.08, 12.61)	7.33^*∗∗*^ (5.37, 10.00)
Intercept (community)	0.12^*∗∗*^ (0.11, 0.14)	0.03^*∗∗*^ (0.02, 0.04)	0.10^*∗∗*^ (0.07, 0.16)	0.03^*∗∗*^ (0.02, 0.06)

*Random effects*				
Observations	11829	11829	11829	11829
Second-level units (community)	427	427	427	427
ICC	0.35	0.31	0.26	0.25
QIC	10235.76	10086.30	9511.01	9615.39
QICu	10226.42	10077.33	9460.96	9600.36

^*∗∗*^ *p* ≤ 0.01; ^*∗*^ *p* ≤ 0.05; ICC: intracommunity correlation coefficient; QIC: quasi-likelihood information criterion; QICu: the simplified version of the QIC.

## Data Availability

The 2014 Ghana DHS dataset supporting the analysis of this study is available in the DHS repository. The DHS datasets are available for free after a simple registration process. The minimal dataset used to support the findings of this study is included within the supplementary information file.

## Supplementary Materials

Supplementary Materials “MultiLevelData.xlsx” is the minimal dataset supporting the findings of the study.
